# Nutritional Composition, Textural, Histological and Structural Properties of Giant Sea Catfish (*Arius thalassinus*) Roe as Affected by Size

**DOI:** 10.3390/foods15050946

**Published:** 2026-03-07

**Authors:** Raj Kumar John Kumar, Suriya Palamae, Mallikarjun Chanchi Prashanthkumar, Watcharapol Suyapoh, Pornpot Nuthong, Bin Zhang, Hui Hong, Soottawat Benjakul

**Affiliations:** 1 International Center of Excellence in Seafood Science and Innovation (ICE-SSI), Faculty of Agro-Industry, Prince of Songkla University, Hat Yai 90110, Songkhla, Thailand; johnrajj777@gmail.com (R.K.J.K.); suriya.pal@psu.ac.th (S.P.); mallikarjun.chanchi@gmail.com (M.C.P.); 2 Faculty of Veterinary Science, Prince of Songkla University, Hat Yai 90110, Songkhla, Thailand; watcharapol.s@psu.ac.th; 3 Office of Scientific Instrument and Testing, Prince of Songkla University, Hat Yai 90110, Songkhla, Thailand; pornpot.n@psu.ac.th; 4 Zhejiang Provincial Key Laboratory of Health Risk Factors for Seafood, College of Food and Pharmacy, Zhejiang Ocean University, Zhoushan 316022, China; zhangbin@zjou.edu.cn; 5 Beijing Laboratory for Food Quality and Safety, College of Food Science and Nutritional Engineering, China Agricultural University, Beijing 100083, China; hhong@cau.edu.cn; 6 BioNanocomposite Research Center, Department of Food and Nutrition, Kyung Hee University, 26 Kyungheedae-ro, Dongdaemun-gu, Seoul 02447, Republic of Korea

**Keywords:** nutrition, fatty acid profile, protein, microstructure, histology

## Abstract

Fish roe is consumed in different forms, e.g., caviar. The large and firm spherical roe from giant sea catfish (GSC, *Arius thalassinus*), which have a high price, are popular in some countries, like Thailand. However, the information on their nutrition and properties is scarce. Roe of different sizes from GSC, including medium (GSC-M), large (GSC-L), and extra-large (GSC-XL) sizes, were rich in protein (29.52–32.70%), fat (4.07–5.65%), and essential amino acids, particularly leucine and lysine. Vitelline was the major protein in GSC roe. Polyunsaturated fatty acids, including eicosapentaenoic acid and docosahexaenoic acid, were abundant, although GSC-M showed lower PUFA content (21.91%) than GSC-L and GSC-XL (25.56–25.94%). No significant differences in texture property were found between sizes, despite the microstructural and histological differences. Larger voids and strands were found with augmenting size, while GSC-L showed greater membrane thickness (133.55 µm). FTIR spectra confirmed the presence of peptide and ester bonds associated with proteins and triacylglycerols, respectively. GSC-L had the highest cholesterol content (651.2 mg/100 g), whereas GSC-M showed the highest α-tocopherol level (1.64 mg/kg). Phosphorus was the dominant mineral (3473–3894 mg/kg), followed by calcium and other minerals. Hence, the roe from GSC, regardless of size, possess high nutritive value and could be used as a wholesome marine food or functional ingredient.

## 1. Introduction

In recent decades, the utilization of marine resources for human consumption has progressively increased worldwide, primarily as a potential source of nutrients associated with health promotion [1]. Fish roe are rich in high-quality proteins, phospholipids, bioactive peptides, and long-chain n-3 polyunsaturated fatty acids (PUFAs), particularly DHA and EPA, which contribute to cardiovascular and neurological health benefits [2]. Furthermore, valorization of underutilized fishery products, e.g., roe is becoming increasingly important for both aquaculture and fisheries sectors [3]. Fish roe, either fully developed or developing in form, is one of the edible parts of fish [4]. It can be considered a functional food ingredient good for cardiovascular and neurological health, owing to its high content of long-chain n-3 polyunsaturated fatty acids [5]. People often refer to the gonads of female fish as “roe”, and they are valuable in both national and international markets [6]. The texture, color, taste, and chemical composition of roe vary from fish to fish and are affected by the processing methods used [7]. People all over the world consume roe as traditional and gourmet dishes. Roe is served as a delicacy in many parts of the world, namely Korea, Russia, and some parts of Europe [6]. Among roe products, caviar is one of the most expensive cuisines. Thailand exported 6461 kg (~6.46 tonnes) of caviar and caviar substitutes (HS 160430) in 2023, with a total export value of USD 101,120 [8].

Roe are rich in phospholipids, proteins, and bioactive peptides. Free amino acids give them their umami taste [9]. Glutamic acid is the principal amino acid responsible for imparting umami flavor to roe-derived products, enhancing their palatability [9]. The availability of essential amino acids, high protein content, phospholipids, and long-chain polyunsaturated fatty acids (LC-PUFAs), particularly docosahexaenoic acid (DHA; C22:6n-3) and eicosapentaenoic acid (EPA; C20:5n-3), makes fish roe a promising source of essential nutrients [3].

The giant sea catfish (*Arius thalassinus*), which belongs to the family Ariidae, is an important demersal fish that can be found throughout the Indo-Pacific region [10]. This species significantly enhances artisanal and commercial fisheries by supplying edible flesh and valuable by-products, such as skin, bones, and roe [11]. Roe of this species is in high market demand due to its unique texture and high nutritive value. Giant sea catfish roe available in the market vary in size, determining their price. Size is a significant element that influences the physicochemical and nutritional characteristics of fish roe [7]. The texture, form, and function of the roe may be altered by differences in the stage of gonadal development, metabolic activity, and the biochemical buildup of macromolecules like proteins and lipids, as influenced by the size of roe [12]. At the biological level, these properties are strongly influenced by oocyte maturation, which governs metabolic activity, lipid and protein deposition, membrane organization, and microstructural development of the roe. Nonetheless, no information is available on the chemical composition and textural properties in relation to the microstructural or histological structure of roe from this species as affected by varying commercial sizes.

Therefore, this study aimed to investigate the chemical composition and the textural, microstructural, and histological properties of giant sea catfish roe having different sizes. The information gained could be of benefit to underscore the nutritive value and properties influenced by roe size. In addition, it could help select the appropriate methods for processing and preservation.

## 2. Materials and Methods

### 2.1. Chemicals

All reagents used in this work were of analytical grade. The chemicals used were purchased from Sigma-Aldrich (St. Louis, MO, USA). Protein markers (8–220 kDa) were acquired from Colorburst™ Electrophoresis Marker, GE Healthcare, Piscataway, NJ, USA.

### 2.2. Collection and Preparation of Roe Samples

Roes from giant sea catfish (*Arius thalassinus*) with three commercial sizes, including medium (GSC-M), large (GSC-L), and extra-large (GSC-XL), were sourced from a seafood supplier in Lat Yai, Mueang District, Samut Songkhram Province, and transferred to Hat Yai, Thailand, under refrigerated conditions (4 °C) within 8 h. All roe samples collected from fish harvested in the same season, sourced from the same supplier, and only fully developed, fertilizable roe without visible signs of immaturity or degeneration were selected. The samples were packed in polyethylene bags and placed in insulated containers with ice packs. Upon arrival at the laboratory, the diameters of individual roe were measured using a digital vernier caliper. The diameters of GSC-M, GSC-L, and GSC-XL were in the ranges of 1.2–1.3 cm, 1.4–1.5 cm, and 1.6–1.7 cm, respectively. The samples were placed in polystyrene boxes containing crushed ice to lower their temperature. The roes were then cleaned with distilled water, drained, packed in polyethylene bags, and stored at −50 °C to terminate microbial activity and enzymatic breakdown until further analysis.

Roe size was the main experimental factor and was categorized following market-based classification. For each group, the roe from five individual roe sacs were pooled. The roes were removed from the sacs, pooled, mixed well, and used as composite samples. For each size, the samples were randomly separated into three different groups, representing three technical replicates.

### 2.3. Proximate Analysis

Moisture, crude protein, fat, ash, and carbohydrate contents of the roe samples were determined according to AOAC methods [13]. All analyses were performed in triplicate.

### 2.4. SDS-PAGE

Protein patterns of the roe samples were analyzed using SDS-PAGE following the Laemmli method [14], as modified by Palanisamy et al. [15]. Roe samples (2 g) were solubilized using 18 mL of 5% SDS solution (85 °C), followed by homogenization at 8000 rpm for 2 min. The mixture was centrifuged at 8000× *g* for 10 min at 4 °C to remove debris. The protein solution was mixed at a 1:1 ratio with loading buffer containing 20% glycerol, 4% SDS, and 125 mM Tris-HCl (pH 6.8). A total of 30 µg of protein was loaded onto a discontinuous gel composed of a 4% stacking gel and a 12% resolving gel. Electrophoresis was conducted using a mini-PROTEIN II cell (Bio-Rad, Hercules, CA, USA) at a constant current of 15 mA until the tracking dye reached the bottom of the gel. Gels were stained with 0.005% Coomassie Brilliant Blue G-250 prepared in 15% methanol and 5% acetic acid and destained in the same solvent until the background became clear.

### 2.5. Amino Acid Profile Analysis

Free amino acid profiles were analyzed using an automated system at Hitachi-Hightech, Bangkok, Thailand, following a modified version of Singh et al. [16]. Free amino acids were separated by ion-exchange chromatography and detected after post-column derivatization with ninhydrin. Quantification was achieved by comparing peak areas with those of standard amino acid mixtures. To determine tryptophan, the samples were prepared separately using alkaline hydrolysis. Samples were hydrolyzed with 4.2 M NaOH at 110 °C for 16 h under nitrogen, followed by HPLC analysis with fluorescence detection, according to the methods of Spies [17] and Landry et al. [18].

### 2.6. Fatty Acid Profile Analysis

Fatty acids were analyzed as methyl esters (FAMEs) prepared using the two-step transmethylation method as described by Bora et al. [19], involving methanolic-NaOH followed by methanolic-HCl. FAMEs were separated using an Agilent 7890B gas chromatograph equipped with a flame ionization detector (GC-FID; Santa Clara, CA, USA). Fatty acids were quantified and expressed as percentages.

### 2.7. Mineral Analysis

Phosphorus (P), calcium (Ca), magnesium (Mg), sodium (Na), zinc (Zn), iron (Fe), and copper (Cu) contents of giant sea catfish roe were determined by inductively coupled plasma–optical emission spectrophotometer, ICP-OES (Avio 500, Perkin Elmer Instruments, Waltham, MA, USA) following the AOAC method [20]. A sample (0.3 g) was mixed with 5 mL of mixed solutions (HNO_3_:HClO_4_:H_2_O_2_ = 5:1:1, *v*/*v*/*v*) for 10 min at 240 °C using 1500 W. Thereafter, the volume was adjusted to 50 mL with deionized water. The solution was subjected to ICP-OES analysis. Flow rates of argon to plasma, auxiliary, and nebulizer were kept at 10, 0.2, and 0.60 L/min, respectively. The sample flow rate was set at 1.2 mL/min. Mineral concentration in the samples was expressed in mg/kg wet weight.

### 2.8. Cholesterol Analysis

Cholesterol content was determined using gas chromatography with a flame ionization detector (GC–FID; Santa Clara, CA, USA) following alkaline saponification and solvent extraction, based on AOAC Official Method [21] and ISO 12228-1 [22]. Briefly, homogenized roe samples were subjected to alkaline saponification using ethanolic potassium hydroxide under reflux conditions (70–80 °C) to liberate free cholesterol from esterified forms. After cooling, the unsaponifiable fraction was extracted with n-hexane, and the organic phase was collected. The extract was concentrated under reduced pressure and reconstituted in an appropriate volume of solvent prior to GC analysis. Cholesterol separation was performed using gas chromatography equipped with a flame ionization detector (GC–FID). The GC system was equipped with a packed glass column (2.4 m × 3 mm i.d.) coated with 0.5% Apiezon L on 80–100 mesh (Chromosorb W HP). Nitrogen was used as the carrier gas at a flow rate of approximately 50 mL min^−1^. Operating temperatures were set at 275 °C for the injector, 230 °C for the column, and 275 °C for the detector. Hydrogen and air flow rates for the FID were maintained at approximately 35 and 350 mL min^−1^, respectively. Cholesterol was identified by comparing its retention time with that of an external cholesterol standard, which was analyzed under identical conditions. Quantification was performed using an external calibration curve constructed from known concentrations of a cholesterol standard. Results were expressed as mg cholesterol per 100 g of sample.

### 2.9. α-tocopherol Analysis

α-tocopherol content was analyzed using solvent extraction followed by reversed-phase HPLC according to the AOAC method [23] and ISO 9936 [24]. Samples were subjected to saponification with ethanolic potassium hydroxide under a nitrogen atmosphere to prevent oxidative degradation, and α-tocopherol was subsequently extracted using n-hexane. The hexane extract was evaporated at room temperature under nitrogen and re-dissolved in methanol prior to chromatographic analysis. The analysis was conducted using an HPLC system (Shimadzu LC-20 AD, Shimadzu Corporation, Kyoto, Japan) equipped with a fluorescence detector operated at an excitation wavelength of 290 nm and an emission wavelength of 330 nm. The separation was performed on a C18 reversed-phase column (250 × 4.6 mm, 5 µm) at 30 °C, using methanol as the mobile phase under isocratic conditions at a flow rate of 1 mL/min. α-tocopherol was identified by matching retention times with an authentic standard and quantified using an external calibration curve. Method performance was verified through assessment of calibration linearity and injection repeatability. Concentrations were expressed as mg α-tocopherol per 100 g of sample.

### 2.10. Texture Profile Analysis (TPA)

Textural properties were assessed using a TA-XT2 texture analyzer (Stable Micro Systems, Surrey, UK) equipped with a 35 mm cylindrical probe, following Murugesan et al. [25] with slight modifications. Each sample was subjected to a two-cycle compression test, in which the compression level was set at 30%. The two compressions were separated by a 2 s interval, and the test speed was maintained at 0.5 mm/s. The software provided values for hardness, cohesiveness, springiness, gumminess, chewiness, and resilience. All measurements were carried out in triplicate, and the mean values were reported.

### 2.11. Scanning Electron Microscopy

Microstructural observation was done as per the procedure outlined by Gwo [26]. Small pieces of roe were fixed in 2.5% glutaraldehyde in 0.12 M phosphate buffer (pH 7.38) for 4 h, and then transferred to 1% osmium tetroxide for 2 h for post-fixation. After fixation, samples were dehydrated through a graded ethanol series (30–100%). The dried specimens were coated with gold by a sputter coater (SPI, West Chester, PA, USA) and then examined using a Quanta 400-FEG scanning electron microscope (FEI, Hillsboro, OR, USA) to visualize surface morphology.

### 2.12. Histological Measurement

Hematoxylin and eosin (H and E) staining was performed [27]. The important features, such as membrane pigmentation, yolk characteristics, the integrity of membrane layers, and any artifacts present in the samples, were observed. Histomorphometric analysis was conducted on individual roe samples, with three biological replicates per size group. For each biological replicate, one representative histological slide was prepared. Membrane thickness was measured in ten non-overlapping microscopic fields per slide, yielding ten measurements per replicate and a total of thirty measurements per size group. Field selection was performed using a two-step approach. First, measurements were restricted to anatomically comparable regions of the roe membrane, specifically the basoeosinophilic fibrous membrane layer with clearly distinguishable inner and outer boundaries. Second, within these regions, fields were selected using a systematic random sampling strategy. After an initial random starting position, the images were acquired following an alternating image–skip–image pattern along with the curvature of the membrane to ensure non-overlapping fields and uniform spatial coverage of the section. To minimize selection bias, the measurements were distributed across the entire section rather than concentrated in a single localized area. Exclusion criteria were defined beforehand, and fields exhibiting freezing–thawing artifacts, artificial clefts between membrane layers, folds, tears, compression, uneven staining, or unclear structural boundaries were excluded from analysis. Only intact membrane regions with clear delineation were used for thickness determinations. Histomorphometric measurements were performed on calibrated digital images using a known micrometer scale. Membrane thickness was quantified using ruler-based tools in ImageJ software (version 2.3.0/1.53t, National Institute of Health, Bethesda, MD, USA). Image acquisition was carried out at ×20 magnification using a digital video capture system (VDO capture digital camera, ECLIPSE Ni-U, Nikon, Tokyo, Japan). All samples were subjected to identical freezing, thawing, fixation, staining, imaging, and analysis procedures to ensure consistency, reproducibility, and robustness of the quantitative histological data.

### 2.13. FTIR Analysis

A Bruker Vertex 70 FTIR spectrometer (Bruker Co., Ettlingen, Germany) was used to examine the FTIR spectra as detailed by Vijayakumar et al. [28]. The roe samples were freeze-dried for 48 h at −55 °C before being finely powdered for analysis. Using a hydraulic press, the powdered material was combined at a 1:10 (*w*/*w*) ratio with spectroscopic-grade KBr to form translucent pellets. With 32 scans and a resolution of 4 cm^−1^, spectra were acquired in the mid-infrared range (4000–500 cm^−1^). The obtained signal was normalized using an empty cell spectrum as a reference. OPUS 3.0 software (Bruker Co., Billerica, MA, USA) was used to process the data.

### 2.14. Experimental Design and Statistical Analysis

A completely randomized design (CRD) was used for the whole study with roe size as the main experimental factor. Results were expressed as mean ± standard deviation based on triplicate determinations. Normality of data distribution was assessed using the Shapiro–Wilk test, and the homogeneity of variances was evaluated using Levene’s test. One-way ANOVA was performed using SPSS version 27.0 (IBM, Armonk, NY, USA). When significant differences were detected, Duncan’s multiple range test was used for post hoc mean separation. Differences were considered statistically significant at *p* < 0.05.

## 3. Results and Discussion

### 3.1. Proximate Compositions

GSC-M, GSC-L, and GSC-XL roe had protein contents of 32.7 ± 0.92% (82.1 ± 0.95%), 30.8 ± 0.83% (81.2 ± 0.85%), and 29.5 ± 0.81% (80.8 ± 0.88%), expressed on a wet and dry weight basis (Table 1), respectively. Much higher protein content was found compared with lipid content. Eggs generally store proteins and lipids in the yolk to aid in the development of the embryo. The results indicated that those roe had less yolk lipid than protein. Moreover, those structural and functional proteins could represent advanced yolk formation with higher metabolic readiness for roe development. High protein levels in GSC-M roe plausibly showcased a variation in hydration-driven dilution effects, resulting in lower protein concentration in larger oocytes (Wallace et al., Arukwe et al., Bekhit et al.) [29,30,31]. Bekhit [3] mentioned that in many marine and freshwater roe, proteins form the primary structural and enzymatic reserves that are important for early embryonic cell division and chorion formation. According to Phetchthumrongchai et al. [9], the protein contents of dried skipjack tuna (*Katsuwonus pelamis*) fell between 69.3 and 70.5%. The elevated protein levels in roe might also be attributed to a thicker chorion and increased deposition of cytoskeletal proteins such as actin and myosin, which in turn help in blastodisc organization [3].

GSC-M, GSC-L, and GSC-XL roe had fat contents of 5.6 ± 0.71% (14.2 ± 0.94%), 4.4 ± 0.64% (12.5 ± 0.92%), and 4.0 ± 0.52% (12.1 ± 0.68%), based on wet and dry weight, respectively. Notably, protein content was substantially higher than fat content in all roe samples, with GSC-M roe exhibiting the highest levels of both components. When water uptake increases during final oocyte maturation [29], lipid concentration is lowered in larger roe due to the dilution effect. As the size of roe increased, lipid content decreased, indicating the utilization of energy during egg development. According to Bekhit [3], marine fish roe rich in phospholipid-filled yolk fractions, which are naturally enriched with DHA. This correlates with the need for enhanced membrane formation and neural development in larger oocytes. The lipid percentages in the present study were comparable to those reported by Mol et al. [32]. Lipid contents of 15.9 g/100 g roe, 14.7 g/100 g roe, and 14.6 g/100 g roe were found for different types of black caviar, namely Beluga, Imperial, and Osetra, respectively.

Similarly, the carbohydrate contents of GSC-M, GSC-L, and GSC-XL roe were in the range of ~1.4–1.9% (~4.0–4.8%). Fish roe often has a lower carbohydrate content, since most of its energy is stored as protein or fat [33]. Ash (mineral) content was lower in GSC-M than those of GSC-L/GSC-XL (from ~0.6 to 0.9%, wet basis or from 1.9 to 2.3%, dry basis). Roes normally have more yolk protein, while smaller roes have comparatively more stable shell or mineral-rich components [34]. This reduction reflects that as the eggs grow, the mineral components are depleted. Overall, the nutrient profile suggested that protein, fat and carbohydrate varied between roes having different sizes (*p* < 0.05). Nonetheless, ash and carbohydrate contents showed minor diffrences between the three sizes. These variations in chemical compositions might be related to biochemical changes as the eggs proceed through further development or growth.

### 3.2. Protein Patterns

**Figure 1:**
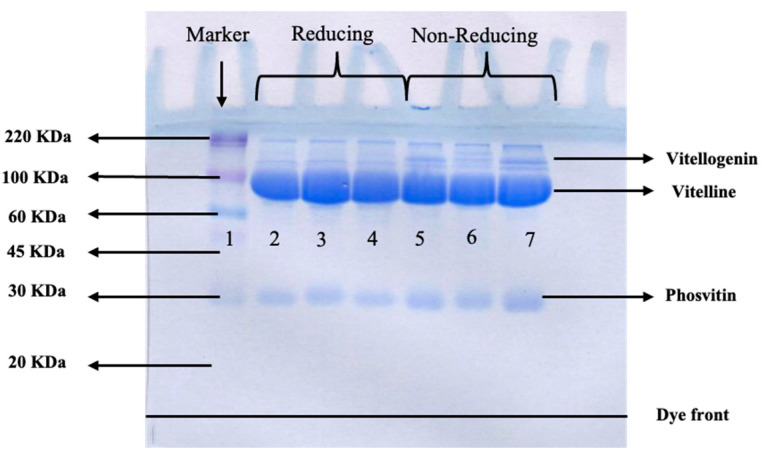
SDS-PAGE profile of roe from giant sea catfish with different sizes under reducing and non-reducing conditions. Lanes: Marker (lane 1), GSC-M (lane 2, 5), GSC-L (lane 3, 6), and GSC-XL (lane 4, 7). GSC-M: giant sea catfish–medium roe with medium size (1.2–1.3 cm in diameter), GSC-L: giant sea catfish–large roe with large size (1.4–1.5 cm in diameter), GSC-XL: giant sea catfish–extra-large roe with extra-large size (1.6–1.7 cm in diameter).

SDS-PAGE under both reducing and non-reducing conditions was carried out to determine the protein patterns of the fish roe (GSC-M, GSC-L and GSC-XL), as shown in Figure 1. Several protein bands larger than 20 kDa were observed, and the general protein bands corresponded well with those typically found in teleost roe [35]. Regardless of sizes, the molecular weights of the proteins were nearly the same for all samples since the roe belong to the same species. Nonetheless, there were minor variations in the protein patterns found under reducing and non-reducing conditions. The protein bands with MW above 100 kDa disappeared under the reducing condition. The results suggested that those proteins, especially those with high MW, were stabilized by disulfide bonds.

Overall, proteins with MW of around 100 kDa, most likely corresponding to vitelline, constituted the major protein, while phosvitin having an MW of 30 kDa was also present in all roes tested, irrespective of size. According to Yoon et al. [36] and Intarasirisawat et al. [37], yellowfin, skipjack, tonggol, and bonito tuna roe contained proteins with MW of 97 kDa known as vitelline. Furthermore, some tuna roe had protein bands with MWs around 32.5 and 29 kDa, which may indicate ovomucoid or phosvitin [35]. Vitellogenin with MW in the range of ~105–108 kDa was also detected in roe of Atlantic halibut [38]. Therefore, the roe had vitelline and phosvitin as the typical proteins, similar to roe from other fish species. These proteins might contribute to the nutritive value and some properties of roe.

### 3.3. Amino Acid Composition

Amino acid compositions of giant sea catfish roe with three different sizes (GSC-M, GSC-L, and GSC-XL) are represented in Table 2. There were differences in amino acid compositions among all the samples. This might result from the roe’s developmental stage. In general, amino acid contents were lower when the size was larger. Among the essential amino acids, leucine was the most prevalent, followed by lysine and valine at quantities of 84.1–98.5 mg/g, 48.3–57.8 mg/g, and 44.15–52.01 mg/g, respectively. For non-essential amino acids, the concentration of glutamic acid/glutamine ranged from 86.6 mg/g in GSC-M to 74.0 mg/g in GSC-XL. In general, the quantities of major amino acids of GSC-L and GSC-XL were not significantly different (*p* > 0.05), but the contents differed from those of GSC-M (*p* < 0.05). Since proteins were precipitated using TCA prior to analysis, non-protein nitrogenous compounds were removed, and the measured amino acids reflect protein-derived fractions [39]. The observed decrease in amino acid content with increasing roe size is consistent with increased oocyte hydration during maturation, resulting in dilution of proteinaceous components rather than selective loss of amino acids.

Differences in amino acid composition among various fish roe could be attributed to several factors. Bekhit et al. [31] demonstrated that the amino acid profile in salmon roe was primarily influenced by the stage of maturity. Similarly, variations in amino acid content in tuna roe might result from differences in habitat, diet, and seasonal changes [37]. Dried tuna roe had the major amino acids including glutamic acid (13.5–14.6 g/100 g protein), leucine (8.06–8.42 g/100 g), aspartic acid (7.8–8.3 g/100 g), alanine (6.6–6.8 g/100 g), and lysine (6.06–6.89 g/100 g) as reported by Phetchthumrongchai et al. [9]. A similar amino acid composition of mullet roe was documented by Lu et al. [40], in which isoleucine, methionine, arginine, and threonine were 46.1 to 39.28 mg/g, 15.9 to 14.1 mg/g, 40.8 to 32.7 mg/g, and 25.2 to 21.9 mg/g, respectively. Tuna roe served as excellent sources of protein, containing amino acids in both mature and immature forms [9]. Additionally, dried tuna roe could be used as a natural food flavor enhancer in food processing industries due to the high levels of glutamic and aspartic acids, which contribute to umami taste [41,42,43]. Branched-chain amino acids such as leucine are known to influence flavor perception both directly and indirectly, serving as precursors for aroma-active compounds formed through enzymatic and non-enzymatic degradation pathways during processing or storage [3,4,6]. In caviar and fish roe products, leucine-derived degradation products have been associated with savory, fatty, and characteristic roe-like notes, contributing to overall flavor complexity [6].

### 3.4. Fatty Acid Profile

The fatty acid profiles of roe possessing varying sizes indicated some differences in saturated fatty acid (SFA), monounsaturated fatty acid (MUFA), and polyunsaturated fatty acid (PUFA) contents (Table 3). SFA levels were highest in the medium (GSC-M) roe (46.94%), while large (GSC-L) and extra-large (GSC-XL) roes showed significantly lower values. Among SFA, palmitic acid (C16:0) was dominant in all size groups, accounting for ~31–33%, followed by stearic acid (C18:0) (~9.9–10.7%).

The palmitic acid abundant in the roe works as an energy source for fish metabolism during maturation and especially during roe development in female fish. Palmitic acid is known to contribute to lipid melting behavior and mouth-coating properties, which are commonly associated with creamy or buttery mouthfeel in lipid-rich foods, including fish roe and caviar products [3,4,6]. GSC-M roe contained a higher MUFA content (29.4%) and oleic acid (C18:1) was dominant. GSC-M had higher oleic acid content (11.8%) compared to those of GSC-L and GSC-XL (~5%). Oleic acid at high concentrations in GSC-M roe might have a vital role in metabolic activities as absorbed energy during the development of the reproductive organs and is crucial for ovarian development and fish growth [3,29,44]. Palmitoleic acid (C16:1) increased with roe size, being lowest in GSC-M and highest in GSC-XL. The level of monounsaturated fatty acid (MUFA) in red salmon roe was 24.4% [32]. The levels of palmitoleic acid in catfish roe ranged from 10.8 to 12.3%, which is in accordance with the findings for mullet roe [40]. The total amount of PUFA increased significantly in GSC-L (25.9%) and GSC-XL (25.5%), while GSC-M showed the lowest content (21.9%). The PUFA concentration was mainly governed by long-chain n-3 fatty acids, EPA (C20:5) and DHA (C22:6). EPA contents in GSC-L and GSC-XL (5.03% and 4.94%) were two-fold higher than that found in GSC-M (2.5%). DHA contents in GSC-L and GSC-XL (~15.6–15.8%) were significantly higher than those of GSC-M (13.6%). Mol and Turan [32] reported DHA contents of approximately 12–16% in various fish roe, including mullet and salmon, which were comparable to the values observed in GSC-L and GSC-XL roe. Roe from Chinook salmon and other salmonid species is known to be rich in DHA due to its high phospholipid content, supporting embryonic and larval development [31,44]. Similarly, skipjack tuna roe contained elevated levels of DHA and EPA, highlighting it as an excellent source of n-3 fatty acids [9,36]. DHA is vital for the healthy development of the retina and brain tissues of fish larvae [5,45]. According to Horrocks et al., Ma et al., and Klaypradit et al. [5,46,47], roe can be used as a functional element since it is a good source of DHA, which is crucial for brain growth and development, learning capacity, and ocular healing from abnormalities. PUFA concentrations were reported to be varied, e.g., pike roe (26.61%) [46], imperial-type caviar (21.04%), and waxed mullet roe (20.9%) [32]. Based on the concentrations of SFA, MUFA, and PUFA, GSC-M differed from GSC-L and GSC-XL (*p* < 0.05). A sample with lower fat may still have higher PUFA when the lipids are rich in phospholipids [32,44,45]. During oocyte development, neutral lipids (mainly triacylglycerols) primarily function as energy reserves, whereas phospholipids are preferentially enriched with long-chain PUFA such as DHA and EPA. Phospholipids are generally essential for membrane biogenesis and embryonic development [29].

### 3.5. Mineral Profile

Mineral contents of GSC roe having various sizes are represented in Table 4. P was the most dominant mineral in the giant GSC roe, followed by Ca, Mg, Na, and Zn, respectively. Fe (<10 mg/kg) and Cu were present in minor amounts. The concentrations of P, Ca, Mg, and Na were significantly higher in GSC-XL (4373 mg/kg, 1203 mg/kg, 166.4 mg/kg, 245.6 mg/kg) compared to those of GSC-M (3894 mg/kg, 1072 mg/kg, 118.2 mg/kg, 86.7 mg/kg) and GSC-L (4260 mg/kg, 1202 mg/kg, 164.6 mg/kg, 223.6 mg/kg) (*p* < 0.05). Zn was found at the highest level in GSC-L (81.82 mg/kg), while GSC-M and GSC-XL had Zn contents of 79.7 mg/kg and 76.02 mg/kg, respectively. Differences were found among all minerals across different roe sizes (*p* < 0.05).

These findings are consistent with previous reports. Eun et al. [48] documented that the predominant minerals found in catfish roe were P, K, Ca, Na, and Mg. Similarly, Bechtel et al. [49] found minerals such as P, K, Na, Ca, Fe, Zn, and Cu in immature pollock roe. P is typically linked to phospholipid concentration and the existence of phosphoproteins [50]. Cu and Fe are categorized as vital trace elements needed for physiological and metabolic processes in marine organisms, including enzyme activity and oxygen transport [50].

Mineral distribution in fish roe is also influenced by maturity. In salmon roe, the distribution of minerals showed higher Ca, Mg, and P contents in mature roe and lower levels of Cu, Fe, Na, and Zn compared with immature salmon roe [31]. Comparable mineral profiles have also been observed in the roe of other fish species. Skipjack tuna (*Katsuwonas pelamis*) roe had phosphorus concentrations of approximately 405 mg/100 g, while potassium and sodium also presented at high levels, highlighting a similar high-mineral pattern in marine fish roe [36].

Fish roe generally contains significantly higher content of most minerals when compared with fish muscle: Zn, Fe, and Cu contents were higher than those in muscle by 11-, 5-, and 3-fold, respectively. Ca content was higher than Mg by 2-fold and higher than Na by 1.5-fold. These minerals are crucial for human health, such as bone mineralization (Ca, P, Mg), oxygen transport and energy metabolism (Fe, Cu), antioxidant defense and immune function (Zn), and electrolyte balance (Na, K) [51,52,53]. For children, roe from GSC can serve as an excellent source of essential minerals (Mg, Zn, Fe, and Cu). A 100 g portion of fish roe can supply approximately 29% of the population reference intake (PRI) of Zn for women, 23% for men, and up to 46% for children. The same portion can provide 18% of the adequate intake (AI) of Mg for women, 16% for men, and 24% for children, emphasizing the nutritional importance of roe as a concentrated and valuable source of essential minerals during growth and development of the human body [31,52]. Consequently, the nutritional implications discussed herein should be interpreted with caution, considering portion size, overall diet, and population-specific health considerations.

### 3.6. Cholesterol Content

The cholesterol contents of GSC roe with different sizes are presented in Table 4. There were differences (*p* < 0.05) among roe of various sizes. The cholesterol content was lowest in medium-sized roe compared to larger ones. GSC-L roe exhibited the highest level of cholesterol (651.2 mg/100 g), whereas the cholesterol contents of GSC-M and GSC-XL were 394.9 mg/100 g and 411.1 mg/100 g, respectively. The observed variation indicated that cholesterol accumulation was more dominant in larger roe.

According to Iwasaki and H.R. [54], 25 different kinds of fish had cholesterol levels ranging from 250 to 650 mg/100 g. In addition, Eun et al. [48] found that channel catfish roe had a cholesterol content of 639 mg/ 100 g. Waxed, salted, and dried mullet (*Mugil cephalus*) roe had a cholesterol level of 387.5 mg/100 g [55]. According to Bekhit et al. [56], raw roe from tongol, bonito, and hoki had lower cholesterol levels of 172 mg/100 g, 122 mg/100 g, and 94 mg/100 g, respectively. Variations in species, reproductive stage, lipid composition, and processing techniques could contribute to these variations in cholesterol concentration across fish roe [57].

Fish roe is a biologically rich matrix in which cholesterol plays a crucial physiological role, particularly in embryonic development, such as cell membrane formation, steroid hormone synthesis [58], lipid transport, and reproduction [59]. The elevated cholesterol content in fish roe may have functional relevance as an essential precursor for the synthesis of steroid hormones such as cortisol, aldosterone, estrogen, progesterone, and testosterone [60]. From a nutritional perspective, dietary cholesterol in fish roe is known to support cardiovascular health, neural development, and gut microbiota functions [2,61,62]. Moreover, dietary cholesterol from marine roe has been reported to have a less pronounced effect on serum cholesterol levels when consumed as part of a diet rich in n-3 fatty acids and bioactive lipids, such as those present in fish roe [61]. The World Health Organization (WHO) and FAO previously suggested a prudent dietary cholesterol intake of less than 300 mg/day for the general adult population, a value that is still widely used as a reference in nutritional studies [63]. The high cholesterol content indicates that fish roe should be consumed in moderate portion sizes and as an occasional food rather than as a staple. It is thought to be nutritionally beneficial when consumed in small amounts and as part of a diet high in unsaturated fats [61,64]. Moreover, dietary cholesterol affects the quality of sperm physiology and reproductive outcomes in humans [65]. Hosomi et al. [58] documented the need for dietary cholesterol in the synthesis of ovarian sex hormones and follicular development.

### 3.7. α-tocopherol Composition

The α-tocopherol contents of GSC roe of different sizes are tabulated in Table 4. The α-tocopherol content varied among the different sizes (*p* < 0.05). Larger roe contained lower quantities of α-tocopherol. GSC-L and GSC-XL had α-tocopherol of 1.4 mg/100 g and 1.2 mg/100 g, respectively. However, medium-sized roe (GSC-M) displayed a higher level of α-tocopherol (1.6 mg/100 g). These results demonstrated that roe size influenced α-tocopherol levels in GSC roe. Lipid-soluble antioxidants like α-tocopherol are required during specific stages of gonadal development [57].

The most significant lipid-soluble antioxidant that affects the oxidative stability of lipids in fish and fish roe is α-tocopherol [61]. It enhances the shelf life of roe products by preventing polyunsaturated fatty acids from oxidative deterioration. The α-tocopherol levels observed in GSC roe were lower than those reported for roe from some marine species such as vendace, whitefish, and Baltic herring, which had α-tocopherol concentrations of 8.9 mg/100 g, 17.3 mg/100 g, and 5.04 mg/100 g, respectively [66]. Nevertheless, comparable retention of α-tocopherol was reported in processed roe, with levels of approximately 9.07 mg/100 g [67].

Notably, α-tocopherol plays a vital role in protecting cell membranes from oxidative stress [68,69], supporting immune function [69], and reducing inflammation in humans [70]. The recommended dietary allowance (RDA) for α-tocopherol is 15 mg/day for adults, and consumption of fish roe can contribute meaningfully to meeting this requirement, particularly when included as part of a balanced diet rich in marine lipids [51,71]. Dysken et al. [72] documented that appropriate supplementation of α-tocopherol can prevent and treat Alzheimer’s disease. In addition, α-tocopherol preserves cardiac function by reducing oxidative stress and inflammation in ischemic injuries [70].

### 3.8. Texture Profile

The GSC-M roe had the highest hardness (3.5 ± 0.94 N) compared with GSC-L (2.7 ± 0.91 N) and GSC-XL (2.8 ± 0.86 N) (Table 5). However, there was no significant difference (*p* > 0.05) in hardness among roe of various sizes. Nowak et al. [73] stated that a sample’s solid composition or the viscosity of amorphous particles affect hardness and mouthfeel. Similarly, springiness, representing the ability to recover after compression, was similar for all roe of different sizes (*p* > 0.05). In addition, similar cohesiveness was also found among roe of different sizes. The reduced value of cohesiveness suggests weaker internal resistance to deformation, since cohesiveness is linked to the internal bonding strength of gelled systems [12,74,75]. For gumminess, chewiness, and resilience, no significant differences (*p* > 0.05) were found among all roes. The formation of protein cross-links might contribute to the aforementioned textural attributes [76]. The similar textural properties across sizes were linked to conserved inner membrane thickness and matrix structure of different roes, which might be related to similar hardness and other textural attributes [77]. Roe size did not exert a statistically significant effect on the measured textural parameters. Further studies with increased sample size and statistical power are required to confirm subtle mechanical differences.

### 3.9. Microstructure of Roe

**Figure 2:**
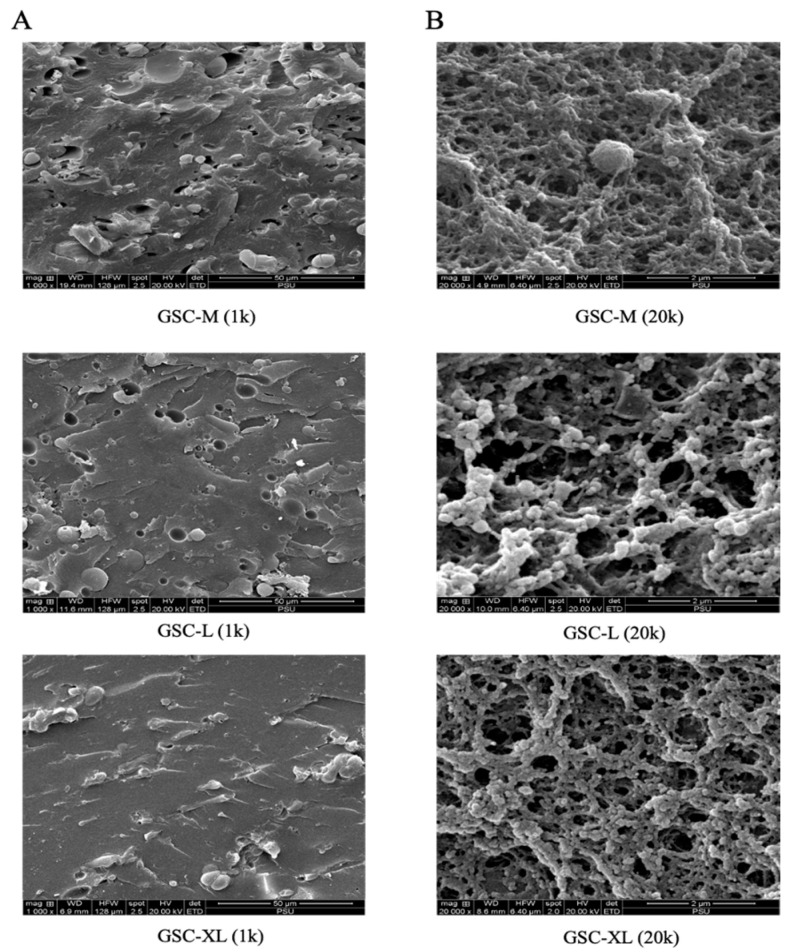
SEM micrographs of giant sea catfish roe with different sizes. Magnifications: low (1000×) (**A**) and high (20,000×) (**B**), respectively. Key: see Figure 1 caption.

The SEM micrographs at 1000× (Figure 2A) clearly illustrate the appearance of voids and some globules in the structure. Slight differences in micro-features were obtained among different roe sizes, providing qualitative insight into the internal organization of the roe matrix. SEM images at 20,000× (Figure 2B) revealed that GSC-M eggs exhibited a denser and more continuous protein matrix with some globules, mainly fat globules. On the other hand, GSC-L and GSC-XL had larger voids and larger strands in the network. The strands with large sizes might provide strength to the network, even though the voids were large. As a consequence, the microstructure did not have a profound impact on the textural properties of roe having various sizes. It was documented that the subtle loosening in larger eggs likely reflects higher PUFA content in larger roe and membrane remodeling during maturation [3]. Overall, SEM micrographs revealed a compact protein–lipid network in all roe of different sizes. Similar structure–texture relationships have been reported in fish eggs, where membrane architecture rather than egg diameter governs mechanical properties [3,78].

### 3.10. Histological Features

**Figure 3:**
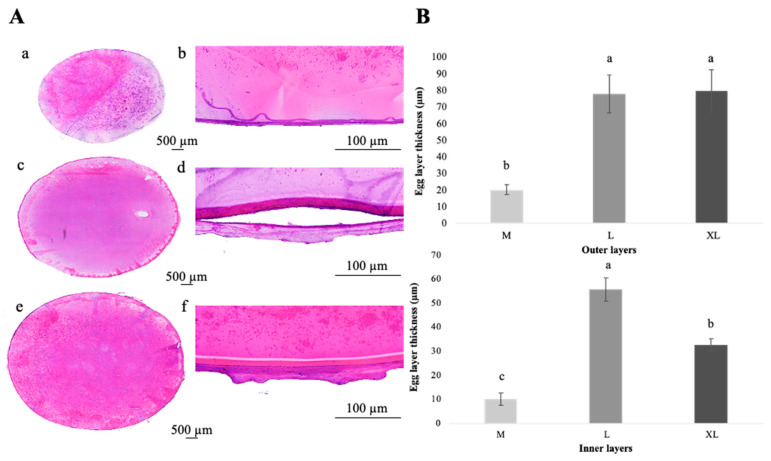
Histological features of roe (**A**) and layer (outer and inner) thickness (*p* < 0.05, according to Duncan’s multiple range test) of giant sea catfish roe with different sizes (**B**). Panels a–b, c–d, and e–f: GSC-M-, GSC-L-, and GSC-XL-sized roe, respectively. Panels a–f: histological structure of the roe membrane layers at low magnification and higher-magnification views. Different lowercase superscripts denote significant differences (*p* < 0.05). Key: see Figure 1 caption.

Roe of three giant sea catfish sizes, including GSC-M, GSC-L, and GSC-XL, were compared histometrically for egg membrane thickness, and the results consistently showed structural variations associated with egg size (Figure 3A,B). The histological structure of the eggs at low magnification demonstrated the progressive increase in egg diameter, yolk content, and membrane development with increasing egg size (Figure 3A: panels a, c and e). At higher-magnification, distinct differences in thickness were found between sizes (Figure 3A: panels b, d, and f). The medium-sized eggs exhibited a thin and compact dual-layered membrane with closely apposed outer and inner lamellae (Figure 3A: panels a, b). The large-sized eggs showed a substantially thicker and more defined membrane structure, suggesting active deposition of proteinaceous and lipid materials during maturation (Figure 3A: panels c, d). The extra-large-sized eggs displayed a markedly thick and stratified membrane with increased density and heterogeneity in the outer layer, reflecting advanced oocyte development and structural reinforcement prior to spawning (Figure 3A: panels e, f).

The thickness of the exterior and inner membranes, as well as the total membrane thickness, were measured in micrometers (µm) under a microscope. The outer layer is generally flexible and depends on egg size and maturation, while the inner layer is conserved and contributes to structural protection of the yolk [30,78]. From Figure 3A, panel c, the amount of water in the yolk region might have formed artificial vacuoles during thawing. In Figure 3A, panel d, the artificial cleft between the inner and outer membrane layers was believed to result from repeated contraction and expansion due to melting and thawing of samples. Overall, the histological findings represented membrane rupture, membrane thickness, and fluid infiltration in the roe.

For medium-sized eggs, the inner membrane was between 7.3 and 18.7 µm, averaging 10.1 ± 2.56 µm, whereas the outer membrane varied from around 15.5 to 28.9 µm, averaging 20.3 ± 2.99 µm. Comparatively thin and compact layers of smaller-sized eggs or early development were evident from the total membrane thickness of 30.5 ± 3.79 µm found in GSC-M.

The large-sized eggs showed a noticeable rise in both layers, with an inner membrane thickness of 55.6 ± 4.80 µm, whereas the outer membrane thickness varied from 59.08 to 96.30 µm, with an average of 77.8 ± 11.5 µm. The overall membrane thickness increased by more than four-fold to 133.5 ± 11.7 µm compared to medium-sized eggs. It was assumed that the larger yolk showed higher metabolic reserves, ensuring improved protection and biochemical deposition during oocyte maturation. The content and structure of membranes are affected by fish oocyte size and maturation stage [30].

Both membranes were much thicker and more structurally complex for GSC-XL eggs. The inner membrane had a mean thickness of 32.6 ± 2.55 µm, whereas the outer membrane varied from 66.6 to 105.9 µm, averaging 79.7 ± 12.76 µm. The average total membrane thickness was 112.3 ± 11.25 µm, indicating that the GSC-XL eggs displayed a more variable and heterogeneous structure, possibly because of postovulatory modifications or hydration-related changes during late maturation [29,30], even though the total thickness was slightly less than that of the GSC-L eggs. A thicker outer membrane may lessen mechanical damage, predation, or microbial intrusion, since larger eggs frequently have larger yolk reserves, slower development, and may be exposed to the environment for a longer period [79]. Despite variations in outer membrane thickness, the conserved inner envelope might have contributed to similar textural properties across roe sizes. Although maturation stage was not directly classified histologically, the consistent and progressive trends across roe size categories suggested that size could serve as a practical proxy for developmental progression in commercially harvested roe.

Overall, histological results showed that the thickness of the egg membrane, especially in the outer layer, was crucial for both selective permeability and mechanical protection. The chorion, also known as the egg coat, has a variety of purposes in teleost fishes, including the defense against microbial infection, selective permeability (i.e., ion and water exchange), and mechanical protection of the developing embryo [78,79]. For instance, Pérez-Atehortúa et al. [79] pointed out that chorion thickness was associated with eggs’ susceptibility to infections, such as *Saprolegnia* spp. While the outer membrane dynamically adjusts to sustain the greater egg volumes and possible environmental stresses associated with advanced egg size classes, the relatively constant inner membrane thickness across groups indicates that its structural function is retained. In general, the inner membrane thickness varied to a lesser degree, and thickness was lower in the GSC-XL group than in the GSC-L group. The finding suggested that the outer layer was more malleable and adapted to the size and maturation stage of the egg, while the inner envelope layer showed a more conserved structural/functional role (such as maintaining basic vitelline envelope integrity) [78]. Although microstructural and histological differences were observed among roe sizes, these characteristics did not translate into mechanical textural changes (Table 5).

### 3.11. FTIR Spectra

**Figure 4:**
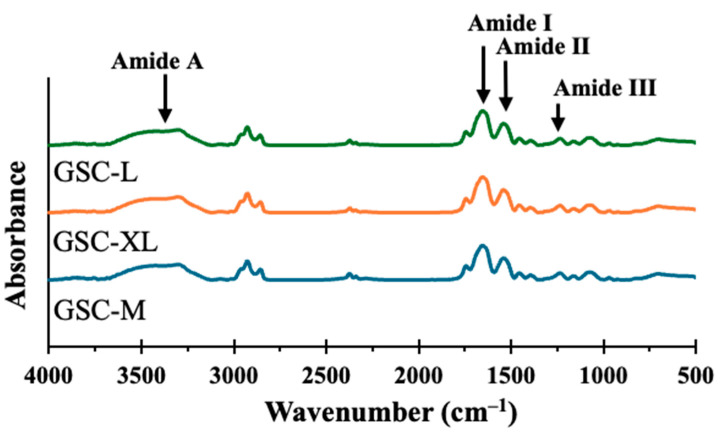
Fourier transform infrared (FTIR) spectra of giant sea catfish roe with different sizes. Key: see Figure 1 caption.

Similar functional groups were revealed in the FTIR spectra of GSC-M, GSC-L and GSC-XL roe samples. Nonetheless, some differences in band intensities reflected biochemical variations between sizes. All three giant sea catfish roes have a pronounced band between 3300 and 3400 cm^−1^, which corresponds to N-H stretching vibrations (amide A) (Figure 4). This peak represents hydrogen bonding within peptide structures. Lipid C-H stretching is indicated by two peaks in the range of 2850–2920 cm^−1^ (presence of triglycerides). C=O stretching and N-H bending vibrations of peptide linkages, which are characteristic of protein secondary structures including α-helices and β-sheets, were reflected by prominent absorption bands at about 1650 cm^−1^ (amide I) and 1540 cm^−1^ (amide II), respectively [80]. A weaker band was observed between 1230 and 1300 cm^−1^ (amide III), resulting from C-N stretching and N-H deformation, representing the proteinaceous composition of the roe matrix [80]. Tiny peaks in the range of 1000–1150 cm^−1^ (confirming ester linkages) suggested the existence of phospholipids and other biological components [81].

A higher level of secondary structural organization or the exposure of peptide bonds was evident from the FTIR spectra of roe samples. This represents increased protein packing or stronger intermolecular interactions, supported by a comparatively tighter or more ordered secondary structure. Hence, the findings suggested that the structural arrangement of proteins and lipids corelated with the FTIR spectra.

## 4. Conclusions

The roe of giant sea catfish had protein as the major component, followed by fat, and overall, medium-sized roe had higher protein content but lower fat content than the larger sizes. In addition, roe had high concentrations of essential amino acids like leucine and lysine. Unsaturated fatty acids (MUFA and PUFA), particularly EPA and DHA, were found in all three sizes of roe, but the larger sizes showed higher contents. Higher levels of Ca, P, and Mg were found in roe with larger sizes. Medium-sized roe had lower cholesterol content but showed higher α-tocopherol than the larger sizes. Roe of different sizes had similar textural properties; however, slightly different microstructural and histological images were found. Collectively, GSC roe could serve as a nutritive diet, in which the target nutrients may vary depending on size. GSC roe hold strong potential for value-added food and nutraceutical applications due to the presence of health-promoting nutrients. Size-specific utilization could be categorized based on the dominant composition. Since roe are perishable, the use of safe preservatives along with potential non-thermal processing technologies should be further investigated to prolong shelf life and ensure the safety of giant sea catfish roe.

## Figures and Tables

**Table 1:** Proximate composition of giant sea catfish roe with different sizes.

Compositions	Content (%, Wet Weight Basis)		
	GSC-M	GSC-L	GSC-XL
Moisture	60.19 ± 0.53 ^b^	61.04 ± 0.95 ^b^	63.47 ± 1.06 ^a^
Protein	32.70 ± 0.92 ^a^	30.86 ± 0.83 ^b^	29.52 ± 0.81 ^c^
Fat	5.65 ± 0.71 ^a^	4.49 ± 0.64 ^b^	4.07 ± 0.52 ^b^
Carbohydrates	1.91 ± 0.36 ^a^	1.64 ± 0.31 ^a^	1.46 ± 0.28 ^a^
Ash	0.55 ± 0.05 ^b^	0.87 ± 0.09 ^a^	0.96 ± 0.16 ^a^
Content (%, Dry Weight Basis)			
Protein	82.10 ± 0.95 ^a^	81.20 ± 0.85 ^b^	80.82 ± 0.88 ^c^
Fat	14.01 ± 0.94 ^a^	12.54 ± 0.92 ^b^	12.15 ± 0.68 ^b^
Carbohydrates	4.50 ± 0.91 ^a^	4.20 ± 0.8 ^a^	4.00 ± 0.64 ^a^
Ash	1.38 ± 0.13 ^b^	2.23 ± 0.23 ^a^	2.63 ± 0.44 ^a^

Values are presented as mean ± standard deviation (*n* = 3). Different lowercase superscripts in the same row denote significant differences (*p* < 0.05) according to Duncan’s multiple range test. Abbreviations: GSC-M: giant sea catfish–medium roe with medium size (1.2–1.3 cm in diameter); GSC-L: giant sea catfish–large roe with large size (1.4–1.5 cm in diameter), GSC-XL: giant sea catfish–extra-large roe with extra-large size (1.6–1.7 cm in diameter).

**Table 2:** Essential and non-essential amino acid composition of giant sea catfish roe of different sizes.

Essential Amino Acids	Content (mg/g)		
	GSC-M	GSC-L	GSC-XL
Leucine (Leu)	35.03 ± 0.54 ^a^	33.20 ± 0.35 ^b^	30.97 ± 0.05 ^c^
Lysine (Lys)	20.54 ± 0.32 ^a^	19.15 ± 0.19 ^b^	17.81 ± 0.02 ^c^
Valine (Val)	18.48 ± 0.23 ^a^	17.58 ± 0.07 ^b^	16.25 ± 0.09 ^c^
Isoleucine (Ile)	16.38 ± 0.26 ^a^	15.47 ± 0.23 ^b^	14.46 ± 0.01 ^c^
Phenylalanine (Phe)	9.22 ± 0.08 ^a^	8.70 ± 0.03 ^b^	8.08 ± 0.06 ^c^
Threonine (Thr)	8.96 ± 0.35 ^a^	8.56 ± 0.04 ^b^	8.05 ± 0.06 ^c^
Histidine (His)	6.33 ± 0.04 ^a^	5.90 ± 0.04 ^b^	5.44 ± 0.00 ^c^
Methionine (Met)	5.68 ± 0.07 ^a^	5.62 ± 0.01 ^a^	5.21 ± 0.00 ^b^
Tryptophan (Trp)	1.42 ± 0.05 ^a^	1.28 ± 0.04 ^b^	1.14 ± 0.03 ^c^
Non-Essential Amino Acids			
Glutamic acid (Glu)/ Glutamine (Gln)	30.78 ± 0.86 ^a^	29.32 ± 0.19 ^b^	27.27 ± 0.14 ^c^
Alanine (Ala)	19.68 ± 0.36 ^a^	18.74 ± 0.20 ^b^	17.60 ± 0.01 ^c^
Aspartic acid (Asp)/ Asparagine (Asn)	17.96 ± 0.36 ^a^	16.99 ± 0.02 ^b^	15.81 ± 0.09 ^c^
Arginine (Arg)	14.51 ± 0.12 ^a^	13.58 ± 0.12 ^b^	12.50 ± 0.01 ^c^
Serine (Ser)	12.24 ± 0.46 ^a^	11.93 ± 0.14 ^b^	11.03 ± 0.10 ^c^
Proline (Pro)	11.48 ± 0.30 ^a^	10.83 ± 0.12 ^b^	9.53 ± 0.09 ^c^
Tyrosine (Tyr)	6.77 ± 0.07 ^a^	6.48 ± 0.03 ^b^	6.06 ± 0.06 ^c^
Glycine (Gly)	6.51 ± 0.10 ^a^	6.31 ± 0.06 ^b^	5.83 ± 0.01 ^c^
Cysteine (Cys)	0.64 ± 0.01 ^c^	0.72 ± 0.01 ^b^	0.73 ± 0.004 ^a^

Values are presented as mean ± standard deviation (*n* = 3). Different lowercase superscripts in the same row denote significant differences (*p* < 0.05) according to Duncan’s multiple range test. Key: see Table 1 caption.

**Table 3:** Fatty acid profile of giant sea catfish roe with different sizes.

Fatty Acids (%)	Formula	GSC-M	GSC-L	GSC-XL
Myristic acid	C14:0	1.23 ± 0.005 ^c^	1.36 ± 0.003 ^b^	1.67 ± 0.05 ^a^
Pentadecanoic acid	C15:0	0.37 ± 0.03 ^a^	0.32 ± 0.003 ^a^	0.22 ± 0.22 ^b^
Palmitic acid	C16:0	32.59 ± 1.02 ^a^	31.90 ± 0.2 ^a^	31.02 ± 1.25 ^a^
Heptadecanoic acid (margaric acid)	C17:0	1.74 ± 0.99 ^a^	1.04 ± 0.69 ^a^	0.82 ± 0.56 ^a^
Stearic acid	C18:0	10.71 ± 0.12 ^a^	9.11 ± 0.07 ^c^	9.93 ± 0.06 ^b^
Arachidic acid	C20:0	0.29 ± 0.008 ^a^	0.22 ± 0.001 ^b^	0.29 ± 0.002 ^a^
∑SFA		46.94 ± 0.57 ^a^	43.96 ± 0.47 ^b^	43.95 ± 0.80 ^b^
Myristoleic acid	C14:1	1.31 ± 0.01 ^a^	0.87 ± 0.003 ^b^	1.02 ± 0.15 ^b^
Palmitoleic acid	C16:1	10.85 ± 0.24 ^b^	11.85 ± 0.76 ^ab^	12.28 ± 0.61 ^a^
Heptadecenoic acid	C17:1	1.10 ± 0.42 ^a^	0.66 ± 0.001 ^a^	0.8 ± 0.12 ^a^
Oleic acid	C18:1	11.81 ± 2.45 ^a^	5.06 ± 0.09 ^b^	5.12 ± 0.07 ^b^
Eicosenoic acid	C20:1	0.99 ± 0.42 ^a^	0.74 ± 0.007 ^a^	0.82 ± 0.41 ^a^
Nervonic acid	C24:1	3.37 ± 0.30 ^a^	2.76 ± 0.54 ^b^	2.43 ± 0.54 ^b^
∑MUFA		29.44 ± 2.84 ^a^	21.94 ± 0.13 ^b^	22.48 ± 1.27 ^b^
Linoleic acid	C18:2	1.08 ± 0.13 ^a^	1.00 ± 0.15 ^a^	1.047 ± 0.18 ^a^
Gamma-linolenic acid	C18:3	1.06 ± 0.41 ^a^	1.23 ± 0.005 ^a^	0.78 ± 0.48 ^a^
Eicosadienoic acid	C20:2	0.35 ± 0.006 ^a^	0.35 ± 0.001 ^a^	0.17 ± 0.48 ^b^
Eicosatrienoic acid	C20:3	0.26 ± 0.07 ^a^	0.35 ± 0.15 ^a^	0.24 ± 0.003 ^a^
Eicosatetraenoic acid	C20:4	2.41 ± 0.008 ^a^	1.97 ± 0.02 ^c^	2.14 ± 0.003 ^b^
Docosadienoic acid	C22:2	0.32 ± 0.02 ^b^	0.43 ± 0.01 ^a^	0.43 ± 0.004 ^a^
Eicosapentaenoic acid (EPA)	C20:5	2.58 ± 0.44 ^b^	5.03 ± 0.060 ^a^	4.94 ± 0.52 ^a^
Docosahexaenoic acid (DHA)	C22:6	13.68 ± 0.13 ^b^	15.58 ± 0.06 ^a^	15:8 ± 0.17 ^a^
∑PUFA		21.91 ± 0.85 ^b^	25.94 ± 0.30 ^a^	25.56 ± 0.59 ^a^

Values are presented as mean ± standard deviation (*n* = 3). Different lowercase superscripts in the same row denote significant differences (*p* < 0.05) according to Duncan’s multiple range test. Key: see Table 1 caption.

**Table 4:** Contents of minerals, cholesterol and α-tocopherol in giant sea catfish roe with different sizes.

Element	Roe Samples		
	GSC-M	GSC-L	GSC-XL
Phosphorus (P) *	3894 ± 111 ^b^	4260 ± 75.83 ^a^	4373 ± 57 ^a^
Calcium (Ca) *	1072 ± 17 ^b^	1202 ± 19 ^a^	1203 ± 34 ^a^
Magnesium (Mg) *	118.2 ± 0.9 ^b^	164.6 ± 2.3 ^a^	166.4 ± 2.7 ^a^
Sodium (Na) *	86.74 ± 1.69 ^c^	223. 6 ± 1.9 ^b^	245.6 ± 3.6 ^a^
Zinc (Zn) *	79.73 ± 0.53 ^b^	81.82 ± 1.36 ^a^	76.02 ± 0.23 ^c^
Iron (Fe) *	3.546 ± 0.15 ^a^	2.771 ± 0.01 ^b^	1.013 ± 0.14 ^c^
Copper (Cu) *	0.846 ± 0.07 ^b^	0.892 ± 0.01 ^b^	1.004 ± 0.14 ^a^

Cholesterol **	394.92 ± 19.7 ^c^	651.20 ± 32.6 ^a^	411.14 ± 20.6 ^b^
α-tocopherol **	1.64 ± 0.08 ^a^	1.43 ± 0.07 ^b^	1.22 ± 0.06 ^c^

Values are presented as mean ± standard deviation (*n* = 3). * mg/kg; ** mg/100 g. Different lowercase superscripts in the same row denote significant differences (*p* < 0.05) according to Duncan’s multiple range test. Key: see Table 1 caption.

**Table 5:** Texture profiles of giant sea catfish roe with different sizes.

Parameters	GSC-M	GSC-L	GSC-XL
Hardness (N)	3.53 ± 0.94 ^a^	2.73 ± 0.91 ^a^	2.87 ± 0.86 ^a^
Springiness	0.99 ± 0.049 ^a^	0.84 ± 0.09 ^a^	0.92 ± 0.35 ^a^
Cohesiveness	0.895 ± 0.24 ^a^	0.849 ± 0.03 ^a^	0.928 ± 0.45 ^a^
Gumminess (N)	2.77 ± 0.23 ^a^	2.42 ± 0.32 ^a^	2.71 ± 0.93 ^a^
Chewiness (N)	2.73 ± 0.07 ^a^	1.78 ± 0.43 ^a^	2.04 ± 0.87 ^a^
Resilience	0.99 ± 0.85 ^a^	0.901 ± 0.94 ^a^	0.967 ± 0.88 ^a^

Values are presented as mean ± standard deviation (*n* = 3). Different lowercase superscripts in the same row denote significant differences (*p* < 0.05) according to Duncan’s multiple range test. Key: see Table 1 caption.

## Data Availability

The original contributions presented in this study are included in the article. Further inquiries can be directed to the corresponding author.
